# A New Synthetic FGF Receptor Antagonist Inhibits Arteriosclerosis in a Mouse Vein Graft Model and Atherosclerosis in Apolipoprotein E-Deficient Mice

**DOI:** 10.1371/journal.pone.0080027

**Published:** 2013-11-04

**Authors:** Frédérique Dol-Gleizes, Nathalie Delesque-Touchard, Anne-Marie Marès, Anne-Laure Nestor, Paul Schaeffer, Françoise Bono

**Affiliations:** Early To Candidate, Sanofi, Toulouse, France; Harvard Medical School, United States of America

## Abstract

**Objective:**

The role of fibroblast growth factors (FGFs) in the development of vascular diseases remains incompletely understood. The objective of this study was to examine the effects of a new small-molecule multi-FGF receptor blocker with allosteric properties, SSR128129E, on neointimal proliferation after a vein graft procedure in mice and on the development of atherosclerosis in atherosclerosis-prone apolipoprotein E (apoE)-deficient mice.

**Methods and Results:**

Vein grafts were performed in 3 month-old male C57BL6 mice. Segments of the vena cava were interposed at the level of the carotid artery. In SSR128129E (50 mg/kg/d)-treated animals, a dramatic decrease in neointimal proliferation was observed 2 and 8 weeks after the graft (72.5 %, p<0.01, and 47.8 %, p<0.05, respectively). Four-week old male apoE-deficient mice were treated with SSR128129E (50 mg/kg/d) for 3 and 5 months in comparison with a control group. SSR128129E treatment resulted in a reduction of lesion size in the aortic sinus (16.4 % (ns) at 3 months and 42.9 % (p<0.01) at 5 months, without any change in serum lipids. SSR128129 significantly reduced FGFR2 mRNA levels in the aortic sinus (p<0.05, n=5-6), but did not affect the mRNA expression levels of other FGF receptors or ligands.

**Conclusion:**

These studies indicate that FGFs have an important role in the development of vascular diseases like atherosclerosis and graft arteriosclerosis. These data suggest that inhibition of FGF receptors by compounds like SSR128129E might be useful as a new therapeutic approach for these vascular pathologies.

## Introduction

Vascular wall inflammation resulting from alterations in lipid metabolism is now recognized to play a central role in the pathogenesis of atherosclerosis [1] and restenosis [2]. Accordingly, the role of inflammatory cytokines and chemokines in the progression of these diseases has been studied extensively in different animal models [3,4]. Growth factors like PDGF and FGF are also prominently expressed in atherosclerotic plaques in humans as well as in experimental animals [5]. Restenosis and post-graft arteriosclerosis are characterized by growth factor-dependent accumulation of extracellular matrix and proliferation of vascular smooth muscle cells (SMCs), following the initial expansion of the vessel intima as a result of the infiltration of inflammatory cells like monocytes. Whereas growth factor-induced SMC proliferation clearly has a deleterious effect in restenosis, the formation of a SMC cap may play a protective role in the stabilization of complex atherosclerotic lesions [6]. Similarly, growth factor-induced angiogenesis in atherosclerotic lesions may either be considered as necessary for plaque perfusion or harmful through plaque destabilization [7–9].

FGF is one of the most potent growth factors for SMCs and endothelial cells and accordingly the role of FGFs as promoters of SMC proliferation has been largely studied *in vitro* and *in vivo*. In particular, smooth muscle cell proliferation after balloon injury or aortic transplant is inhibited by anti-FGF2 antibody or soluble FGFR1, suggesting a direct implication of FGFs in restenosis induced by different mechanisms [10,11]. FGF receptor blockade can therefore be expected to have beneficial effects in these pathologies. In contrast, the role of FGF receptors in the initiation and progression of atherosclerosis is less clear. Although the presence of FGF1 and FGF2 as well as their receptors has been shown on atherosclerotic arteries of rat and human origin [5], only very few studies have addressed the role of these receptors during the early phases of atherosclerosis development. 

In this respect, a recent study using apoE-KO transgenic mice expressing constitutively active FGFR2 in endothelial cells convincingly demonstrated that FGFR2 activation can lead to increased lesion size and smooth muscle cell proliferation [12], suggesting that it would be worthwhile to study FGFR inhibition as a potential treatment of atherosclerotic disease. Actually, the effects of an inhibitor of the tyrosine kinase activity of FGFR1, SU5402, on atherosclerotic lesions of apoE-deficient mice has been reported previously [13]. This compound decreased lesion size, as well as lesion macrophage and smooth muscle cell content. However, SU5402 cannot be considered as a selective FGF-receptor inhibitor, because it inhibits VEGFR2 kinase activity as potently as the activity of FGFR1 [14], and VEGFR2 inhibition has potent anti-atherosclerotic effects [15,16]. We have recently described a selective and potent small molecule FGF receptor antagonist, SSR128129E, which inhibits fibroblast growth factor receptor (FGFR) signaling by binding to the extracellular domain, without affecting orthosteric FGF binding. SSR128129E exhibits allosteric properties, including probe dependence, signaling bias and ceiling effect. SSR128129 is active after oral administration and blocks functional effects of FGF2 like angiogenesis in vitro and in vivo [17]. This compound thus is an ideal tool to further investigate the role of FGF receptor activation in restenosis as well as atherosclerosis development. The effect of SSR128129E was therefore assessed in a mouse model of vein graft arteriosclerosis as well as the apoE-KO model of atherosclerosis.

## Methods

### Mice and Ethics Statement

Male homozygous apoE^-/-^ mice on a C57BL/6J background were obtained from Charles River France at 4-5 weeks of age. All procedures and care of animals were approved by the Animal Care and Use Committee of Sanofi-Aventis Recherche. All animal care programs were accredited by the AAALAC association.

### Vein Graft Model

The procedure used for vein grafts was similar to that described previously by Zou et al. [18].

Briefly, male C57BL/6J mice weighing 30-32 g were anesthetized with ketamine and xylazine (120 mg/kg and 12 mg/kg body wt ip, respectively). The right common carotid artery was dissected free from adhering connective tissue, cut in the middle, and a cuff placed at each free end. The artery was then turned inside out over the cuff and ligated. A vena cava vein from another syngeneic mouse was grafted between the 2 ends of the carotid artery by sleeving the ends of the vein over the artery cuff and ligating them together with an 8-0 suture.

Animals with a successful vein graft were randomized into 2 groups, one with normal chow and one with a diet containing 367 mg of SSR128129E/kg (Bioserv, Frenchtown, USA). This amount of SSR128129 led to an average daily SSR128129 dose of 36±3.3 mg/kg. The average daily dose was lower during the recovery phase after surgery and increased thereafter (Figure S1).

The vein grafts were harvested by cutting off the implanted segments from the native vessels at the cuff end either two or eight weeks after the graft procedure.

After fixation with formaldehyde, the grafts were cut in the middle of the vein segments, dehydrated, and embedded in paraffin. After staining with Masson’s stain [19], proliferation inside the vessel wall was evaluated for each animal by measuring the area of 10 sections of 5 µm thickness, taken at 50 µm intervals, using a computer-assisted image analysis system (MorphoExpert, Explora Nova, La Rochelle, France).

### Mouse model of atherosclerosis

The mice were fed *ad libitum* with a normal mouse chow, or a diet containing 367 mg of SSR128129E per kg (BioServ, Frenchtown, USA). Food intake and weight of each animal were monitored weekly. Accordingly, the average dose of SSR128129 was found to be 43±1.0 and 46±1.0 during 3 months and 5 months of treatment, respectively (Figure S1).After a 5 month treatment period, mice were anaesthetized with sodium pentobarbital (6 mg/mouse; Sanofi Santé Animale, France) and hearts were removed. The hearts were processed as previously described [20]. The extent of atherosclerosis was determined in the aortic root. The upper part of the heart was fixed at 4°C in buffered 4% paraformaldehyde, pH 7.4, snap-frozen, and stained with oil red O. A computer-assisted image analysis system was used to quantify the area of the atherosclerotic lesions within the sections (MorphoExpert, Explora Nova, La Rochelle, France). The areas of the lesions apparent from the oil red O staining were automatically traced, and the total lesion area for each section was calculated. Ten sequential alternate sections from each animal were analysed, and the mean lesion area was calculated for each animal and subsequently for each group.

#### Serum lipid analysis

Total cholesterol and triglyceride levels in serum were determined using automatic enzymatic methods (ABX Diagnostics, France), according to the manufacturer’s instructions.

### Quantitative real-time PCR Analysis

FGFs and FGFRs were analyzed by real-time reverse transcription analysis (RT-PCR) by using RNA samples from aortic sinus of apoE-deficient and control mice. PCR reactions were carried out using Assays-on-demand™ Gene Expression Products (PE Applied Biosystems, Weiterstadt, Germany). Reactions were performed as previously described [21]. The calculations of the initial mRNA copy numbers in each sample were made according to the cycle threshold (CT) method [22] and normalized using TATA-Box binding protein (TBP) mRNA levels.

### Immunohistochemistry and specific staining

Macrophages were detected in paraffin-embedded sections, using a primary rat anti-mouse mac-3 monoclonal antibody (Becton Dickinson Biosciences, France) diluted 1:200 in PBS, followed by a biotinylated secondary antibody and horseradish peroxydase complex (Vectastain, Vector, Burlingame, CA) according to the manufacturer's specifications. Antibody binding was visualized with 3-amino-9-ethyl carbazole (AEC, Zymed,France) and all sections were counterstained for 1 minute with hematoxylin (Zymed, France).

Smooth muscle cells were detected with monoclonal α-actin antibody (diluted 1:200 in PBS; Sigma-Aldrich, St. Louis, MO)) followed by a biotinylated secondary antibody and a horseradish peroxydase complex (MOM, Vector, Burlingame, CA). Antibody binding was visualized with VIP, a peroxydase substrate from Vector (Burlingame, CA). Masson’s trichrome stain was carried out as described by Ganter and Jollés (1970) [19].

FGFR1 was detected with a rabbit polyclonal anti-FGFR1 antibody (diluted 1:1000 in PNS; Abcam, France) followed by a biotinylated secondary antibody and horseradish peroxydase complex (Vectastain, Vector, Burlingame, CA) according to the manufacturer's specifications. Antibody binding was visualized with 3,3'-diaminobenzidine, a peroxidase substrate (DAB, Zymed, France)

### Statistical analysis

Descriptive statistics are given as mean ± SEM.

The hypothesis of normality (Shapiro-Wilk’s test) was checked on raw data. For myointimal proliferation, a log transformation was necessary to correct non-normality.

Student’s t-test was performed for all the analyses of the vein graft model and atherosclerosis model in order to compare the SSR128129E group to the control group. In case of heterogeneity of variance, Satterthwaite’s correction was applied.

For PCR data, a log transformation was applied to all the parameters because of the nature of the data (expressed as fold increase). For all the parameters tested on 6 month-old mice a one-way ANOVA test followed by Dunnett’s test was applied in order to compare each group to the apoE-KO group.

The statistical analyses were performed using SAS V9.2. A p-value lower than 0.05 was considered as significant.

## Results

### SSR128129E inhibits neointimal hyperplasia in a vein graft model

**Figure 1:**
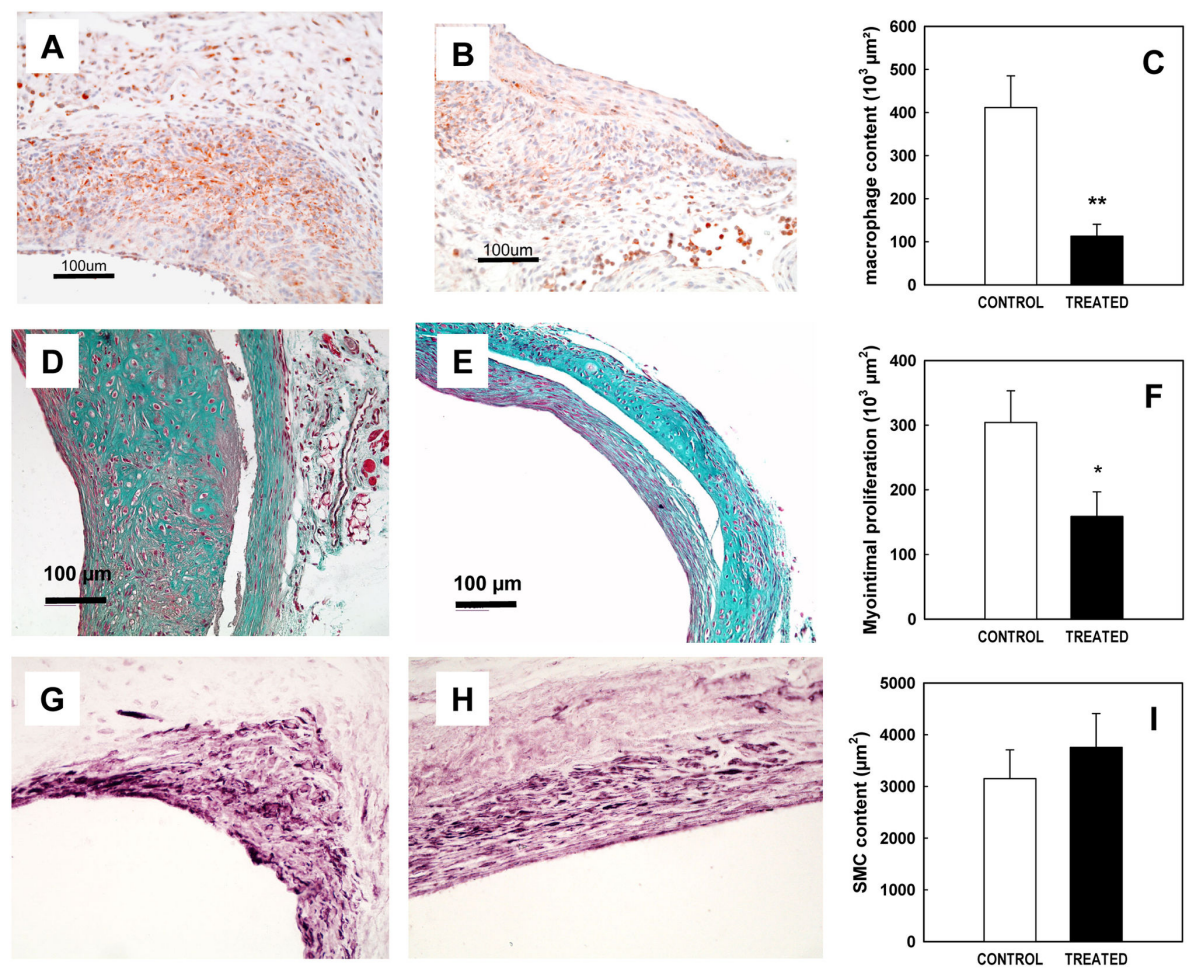
Effect of SSR128129E on neointimal proliferation in the vein graft model. A-C: Effect on lesion macrophage content 2 weeks after surgery Representative sections of vein grafts immunostained for macrophages (mac3) in control (A) and SSR128129E-treated mice (B). The size of the regions showing macrophage infiltration was determined by image analysis (C, n= 4 per group). D-I: Effect on neointimal proliferation 8 weeks after surgery. Representative sections of vein grafts stained by Masson’s trichrome in control (D) and SSR128129E-treated mice (E). The area of neointimal proliferation was determined by image analysis (F, n= 8-11 per group). Smooth muscle content is shown in representative sections of vein grafts labelled for α-actin in control (G) and SSR128129E-treated mice (H). Smooth muscle content as determined by the area of α-actin staining was determined by image analysis (I, n= 8-11 per group).

The FGF receptor antagonist was evaluated in a mouse model of venous bypass graft arteriosclerosis. Two weeks after surgery, control vein grafts showed a large wall expansion essentially due to macrophage infiltration as shown in Figure 1A. Treatment with SSR128129E (50 mg/kg/d) dramatically reduced this proliferation (72.5 % inhibition, p<0.01, n=4, Figure 1B,1C).

 Eight weeks after surgery, controls vein grafts (Figure 1D) showed an important thickening of the vessel wall (surface: 304082 µm^2^ ± 48996), and increased matrix protein accumulation as shown by Masson’s trichrome staining. This thickening was significantly reduced by treatment with SSR128129E (47.8% inhibition, p<0.05, n=8-11, Figure 1 E-F). Interestingly, the percentage of SMCs evaluated by α-actin labeling was unchanged between the placebo and SSR128129E-treated groups (ns, n=8-11, Figure 1 G-I). 

### Effects of SSR128129E on atherosclerosis

In order to determine whether a FGF receptor antagonist could affect atherosclerosis development, one month-old apoE-deficient mice were treated with SSR128129E (50mg/kg/d) for 3 and 5 months.

**Figure 2:**
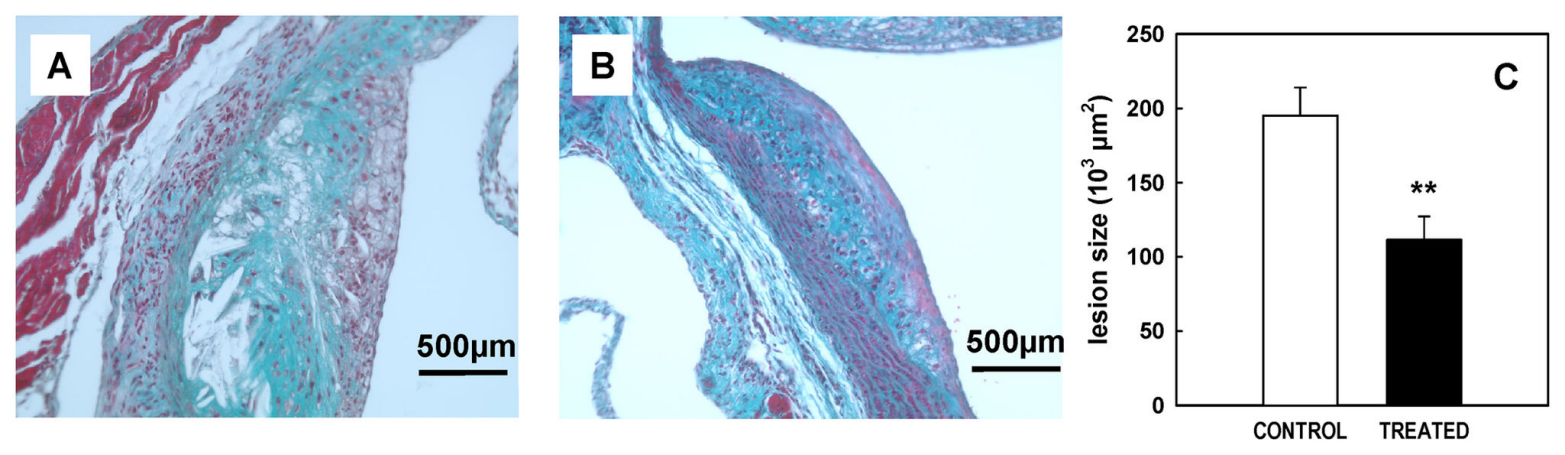
Lesion morphology and size in the aortic sinus of 6 month old apoE-deficient mice. Lesion morphology as shown in representative slices of aortic sinus stained by Masson’s trichrome in 6 month-old control apoE-KO (A) and SSR128129E-treated mice (B). Aortic sinus lesion size was determined by image analysis in 6 month-old apoE-deficient mice (C, n= 8 per group).

**Figure 3:**
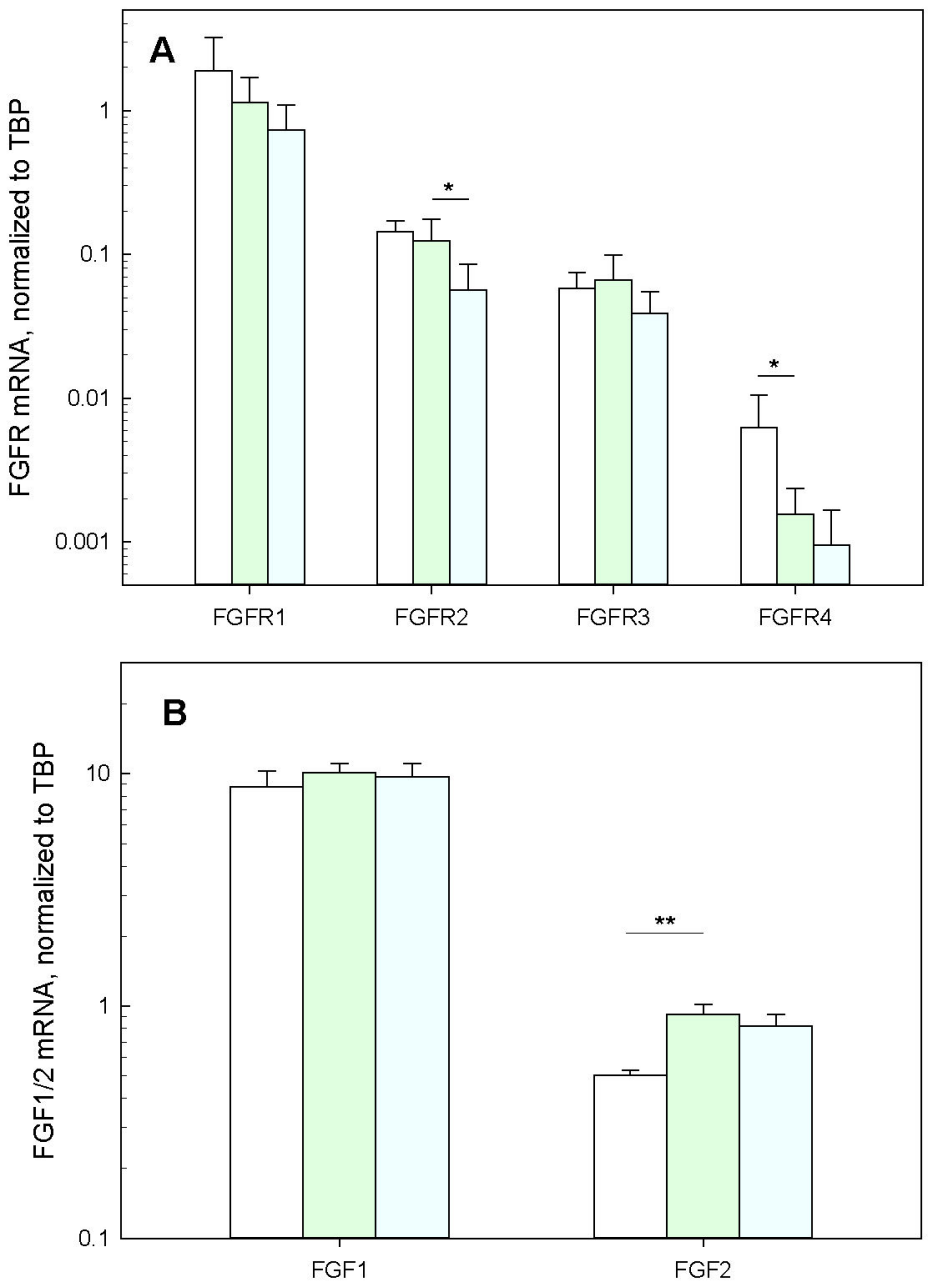
mRNA expression levels of FGF receptors and ligands in the aortic sinus. mRNA levels were determined by quantitative PCR of extracts of the aortic sinus of 6 month-old normal mice (C57BL/6, white bars), apoE-deficient mice (light green bars) and SSR128129-treated apoE-deficient mice (light blue bars). Bars represent the mean±SEM of the data, n=5-6. (*: p<0.05 , **: p<0.01).

After a 3-month period on normal diet, apoE-deficient mice develop fatty streak lesions. Treatment with SSR128129E in the diet slightly reduced these primary lesions (16.4%, ns; data not shown). After 5 months of treatment, control mice showed extensive lesions, intimal thickening and smooth muscle proliferation, as well as appearance of cholesterol clefts (Figure 2A). SSR1282129E-treatment significantly reduced the lesion size (42.9% inhibition, p<0.01, n=8-10, Figures 2B and 2C). The amount of smooth muscle cells within 6 month-old lesions was determined by IHC as described in the previous paragraph. In control animals, the percentage of SMCs within the lesion was 44%. Interestingly, in SSR1218129 treated animals this percentage of SMCs within the lesion was 72 %, showing that the plaque was not only reduced but also richer in SMCs than control plaque. In order to shed some light on the mechanism of action of SSR128129E, the mRNA levels of a number of FGF receptors and ligands, as well as chemokines and cytokines, were determined by PCR on extracts from the aortic root of six month-old wild-type (WT) mice (i.e. C57BL/6) , as well as apoE-deficient mice that had been fed normal chow or chow containing SSR128129E for 5 months. A shown on Figure 3A, aortic roots from normal as well as apoE-deficient mice expressed similar high levels of FGFR1 mRNA, whereas the levels of the other FGF receptors were quite low. The mRNA levels of FGFR4 were significantly decreased in apoE-deficient mice as compared to C57BL/6 mice (p<0.05, n=5-6), although the relevance of this finding is questionable, considering the low absolute expression of FGFR4 mRNA. Treatment of apoE-deficient mice with SSR128129E did not affect the mRNA levels of all the receptors but FGFR2, the levels of which were slightly, but significantly, reduced (p<0.05, n=5-6) (Figure 3A). Regarding FGF-receptor ligands, both FGF1 and FGF2 mRNA were expressed in the aortic root of normal and atherosclerotic apoE-deficient mice. However, lesions from apoE-deficient mice expressed significantly more FGF2 mRNA than aortas from normal mice (p<0.01, n=5-6, Figure 3B). Again, mRNA levels of both FGF1 and FGF2 were very similar between untreated and SSR128129E-treated animals.

**Figure 4:**
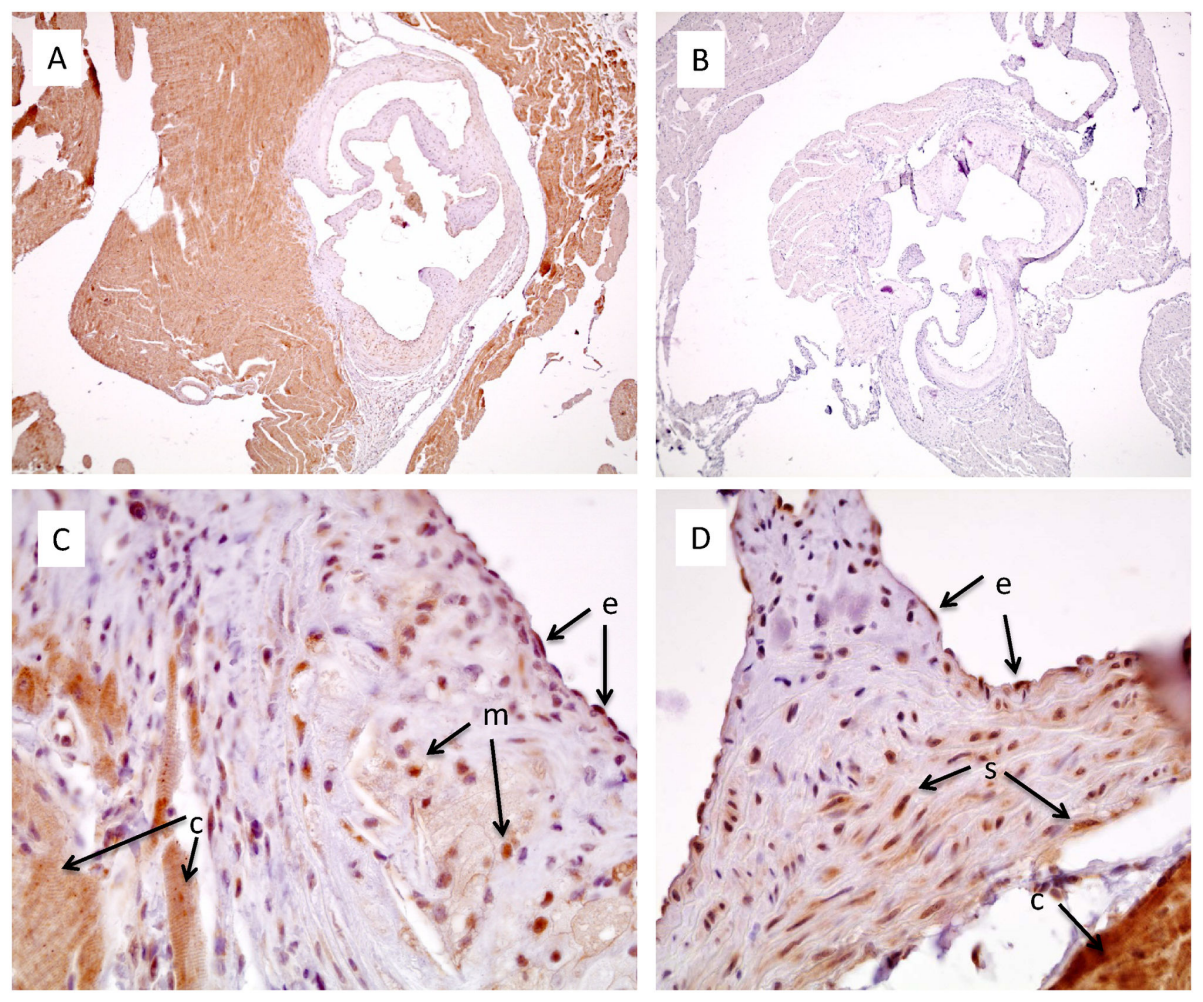
FGFR1 Immunostaining in the aortic sinus of 6 month-old mice. Sections from apoE-deficient (A,B,C) or C57BL/6 mice (D) were immunostained for FGFR1. Omission of the primary antibody led to complete disappearance of the staining (B). The labels next to the arrows indicate the different cell types: e - endothelial cells; m – macrophages; c – cardiomyocytes; s – smooth muscle cells. A 4x objective was used for wide field images (A,B), whereas a 40x objective was used for the high resolution photomicrographs (C,D).

The presence of the FGFR1 was also verified by immunohistochemistry. As shown in Figure 4, the strong staining for FGFR1, particularly in cardiomyocytes, (Figure 4A) disappeared when the primary antibody was omitted (Figure 4B), showing that the staining was specific for the primary antibody. Higher resolution photomicrographs of the normal (Figure 4D) and atherosclerotic (Figure 4C) aortic sinus confirmed that FGFR1 was strongly expressed in cardiomyocytes, but was also present in endothelial cells, smooth muscle cells, and lesion macrophages.

We also determined the mRNA levels of the following chemokines/cytokines in these same samples: CCL2 (formerly called MCP-1), CXCL1 (formerly called KC), CCL5 (formerly called RANTES), CXCL6 (formerly called GCP-2), CD40 and CD40 ligand (also called CD154). Surprisingly, lesioned aortas from apoE-deficient mice did not express higher cytokine mRNA levels than normal aortas from C57BL/6 mice (CCL2 and CXCL6 mRNA levels were actually significantly decreased, p<0.01, n=5-6, data not shown). SSR128129E treatment did not significantly affect the mRNA expression of any of these lesion cytokines (data not shown).

Finally, the assay of serum triglycerides and total cholesterol showed that 6 month-old apoE-deficient mice had higher triglycerides and total cholesterol than C57BL/6 mice, but no difference was observed between control and SSR128129E-treated groups (ns, n=14-15, data not shown).

## Discussion

Two decades ago, FGF was considered a potential key player in atherosclerosis and a possible culprit in the evolution of unstable angina through smooth muscle proliferation and subsequent luminal narrowing [23]. However, the recognition that most coronary events are due to the rupture of thin fibrous caps or plaque erosion, and not to plaques with thick, smooth muscle cell-containing fibrous caps [24,25], cast some doubts on the beneficial effects of FGF receptor inhibition in atherosclerosis. Furthermore, FGF2 has been shown to protect smooth muscle cells from apoptosis [26], and smooth muscle cell apoptosis may play a role in plaque instability [27,28]. Recently however, results from a study using transgenic apoE-deficient mice expressing constitutively active FGFR2 in endothelial cells again suggested that endothelial FGFR2 activation may be deleterious through p21^Cip1^-mediated endothelial cell dysfunction and PDGF-mediated vascular smooth muscle proliferation [12]. It is therefore still not entirely clear whether systemic inhibition of FGF receptors will have protective or deleterious effects in atherosclerosis. 

Regarding systemic FGFR inhibition, one paper described the beneficial effect of the compound SU5402 in the inhibition of the progression of atherosclerosis in apoE-deficient mice. This compound was initially described as a FGFR tyrosine kinase inhibitor, but was also found to be a potent inhibitor of VEGF receptor activation in cells [14,29], and it is therefore difficult to unequivocally ascribe its effects to inhibition of either FGF or VEGF receptor activation.

We have taken advantage of the availability of a new compound, SSR128129E, a selective FGF receptor antagonist, to address the question of the effect of systemic FGF receptor blockade in mouse models of atherosclerosis and restenosis.

As a first step, the effect of SSR128129E was studied in a model of myointimal proliferation induced by replacing one mouse carotid artery by a vena cava from syngeneic animals. This model mimicks the clinical situation of vein graft disease after coronary bypass procedures, giving rise to an initial inflammatory response and subsequent progressive stenosis due to neointimal smooth muscle proliferation [30]. As could be expected, SSR128129E was active in this model, in that it decreased the thickness of the vessel intima, but further immunohistochemical analysis of intimal lesions showed some surprising effects. First, SSR128129E unexpectedly decreased the initial inflammatory component due to macrophage influx into the intima two weeks after the graft procedure. Interestingly, it has recently been reported that intradermal FGF injection leads to the induction of endothelial adhesion molecule expression and subsequent increased leukocyte recruitment into the skin [31]. The effect of SSR128129E could be explained if similar mechanisms played a role in monocyte recruitment to the intima after the vein graft procedure. Similarly, macrophage content of lesions in apo-KO mice expressing constitutively active FGFR2 was increased, also suggesting some role of FGFR2 in macrophage recruitment [12]. A second unexpected finding was related to the effect of SSR128129E on smooth muscle cell proliferation in the neointima. In our hands, the percentage of α-smooth actin-positive area of neointima was similar in control and SSR128129E-treated animals, although the total neointimal area was decreased, suggesting that SSR128129E did not inhibit smooth muscle proliferation, but some other component of neointimal expansion e.g. macrophage accumulation. These results suggest that early inhibition of vein graft inflammation may be the predominant effect of SSR128129.

Considering the impressive effect of SSR128129E on macrophage accumulation early after the vein graft procedure, it was interesting to determine whether SSR128129E would also affect macrophage recruitment to atherosclerotic lesions in apoE-deficient mice. As the role of FGF in atherosclerosis is much less documented than its role in restenosis, we checked the presence of FGF and its receptors in atherosclerotic lesions of apoE-deficient mice and found high expression of FGFR1 and the FGFR ligands FGF1 and FGF2. Whereas FGFR mRNA expression did not change, FGF2 mRNA was significantly upregulated in lesions from 6 month-old mice, suggesting that FGF2 may play some role in lesion growth and that inhibition of FGFR signaling could be effective. These findings corroborate those obtained by Raj et al., who also found the presence of FGF2 and FGFR in the aorta from apoE-deficient mice [13]. Interestingly, SSR128129E treatment slightly, but significantly, reduced the mRNA levels of FGFR2, which has recently been shown to play a role in lesion progression in apoE-deficient mice [12], suggesting that modulation of FGFR2 expression may be a potential mechanism of action of SSR128129E in addition to inhibition of FGFR signaling. However, SSR128129E treatment did not affect the mRNA expression levels of the FGFR ligands FGF1 and FGF2, although a change in FGF2 mRNA would have been expected, considering the decrease in lesion size and the finding that aortic sinus tissue from apoE-deficient mice expressed significantly more FGF2 mRNA than normal aorta from C57BL/6 mice. One explanation may be that the aortic tissue extracts used in our studies were specifically enriched in aortic sinus (where the atherosclerotic lesions are localized) and unavoidably contained a significant proportion of cardiac tissue (as can also be seen in Figure 4A). The well-known high expression of FGFs as well as FGFR1 in cardiomyocytes [32–34] may therefore have masked any changes in lower-expressing lesion cells. A similar reason might also explain why we were unable to detect increases in chemokines between aortic roots from apoE-deficient and C57BL/6 mice, in contrast to our own earlier studies [35]. Whatever the reasons for these discrepancies, the absence of effect of SSR128129E treatment on chemokine/cytokine levels suggests that the effects of SSR128129E are not due to a systemic anti-inflammatory activity.

Unexpectedly, SSR128129E was found to decrease early, macrophage-rich, atherosclerotic lesions only slightly, whereas the compound was much more active against later, more complex lesions. Interestingly, SSR128129E again did not decrease, but rather increased, the percentage of lesion area taken up by smooth muscle cells in these advanced lesions. These data are in sharp contrast to the results described for the FGFR1/VEGFR2 tyrosine kinase inhibitor SU5402, which strongly decreased smooth muscle content of the lesions of apoE-deficient mice in addition to its effect on lesion size. Interestingly, vaccination against VEGFR2 has been shown to decrease lesion size in apoE-deficient [16], as well as LDLR-deficient [15], mice without affecting smooth muscle or collagen content of the lesions. Our data with SSR128129E show that selective inhibition of FGF receptors does not abolish smooth muscle proliferation either in the progression of atherosclerotic lesions or in vein graft arteriosclerosis. Actually, SSR128129E decreased the size of complex lesions and induced smooth muscle cell-rich lesions which can be considered much more stable than the lesions in control apoE-deficient animals. Together with the results from vaccination against VEGFR2 [15], these data suggest that selective inhibition of either VEGFR2 or FGF receptors decreases lesion size without affecting lesion stability. Combined inhibition of VEGF and FGF receptors with SU5402 has potent effects on lesion size, but may also decrease lesion stability through the decreased smooth muscle content of the remaining lesions.

The effects of SSR128129E would not have been expected from in vitro data observed on cultured smooth muscle cells and they therefore suggest that the in vivo effect of FGF receptor inhibition by SSR128129E is not solely related to an effect on smooth muscle cells. One other mechanism could be related to neovascularization which has been reported to play an important role in atherosclerosis, mainly in the unstable plaque phenotype [36]. However, we did not observe any neovascularization in plaques from 4 or 6 month-old apoE-KO mice. Therefore, the hypothesis that SSR128129E could be effective by its potent anti-angiogenic activities [17] cannot be retained to explain our results. SSR128129E probably exerts a regulatory effect on a complex network of FGF-dependent interactions between the different cell types present in restenotic and atherosclerotic lesions. Alternatively, it can be speculated that treatment with SSR128129E, which as an allosteric inhibitor of FGF receptors does not fully and indiscriminately abolish all FGFR signaling [17], may have led to phenotypic modulation of the heterogenous population of plaque smooth muscle cells [37], inhibiting excessive proliferation, but preserving a significant proportion of spindle-shaped, well-differentiated smooth muscle cells in the lesions. This speculation, although attractive, would however have to be substantiated by further mechanistic experiments.

In conclusion, the novel FGF receptor antagonist SSR128129E decreased neointimal proliferation after a vein graft procedure and atherosclerotic lesion progression in apoE-deficient mice. These data suggest that FGF receptor inhibition may have beneficial effects in vascular diseases and highlight that an orally-active small-molecule modulator of FGF receptor tyrosine kinase with allosteric properties may offer novel opportunities to improve anti-atherosclerosis treatment.

## Supporting Information

Figure S1 **Daily dose of SSR128129E as calculated from food uptake in the different models.** The daily dose was determined from the concentration of SSR128129E in the food pellets (367 mg/kg), food intake and animal weight in the vein graft model (A) and during 3 month (B) and 5 month (C) treatment in apoE-deficient mice.
(PDF)
