# Supplementary figures and images for: A New Synthetic FGF Receptor Antagonist Inhibits Arteriosclerosis in a Mouse Vein Graft Model and Atherosclerosis in Apolipoprotein E-Deficient Mice
Source: PLoS One. 2013 Nov 4;8(11):e80027. doi: 10.1371/journal.pone.0080027 (PMC3817113; doi:10.1371/journal.pone.0080027)

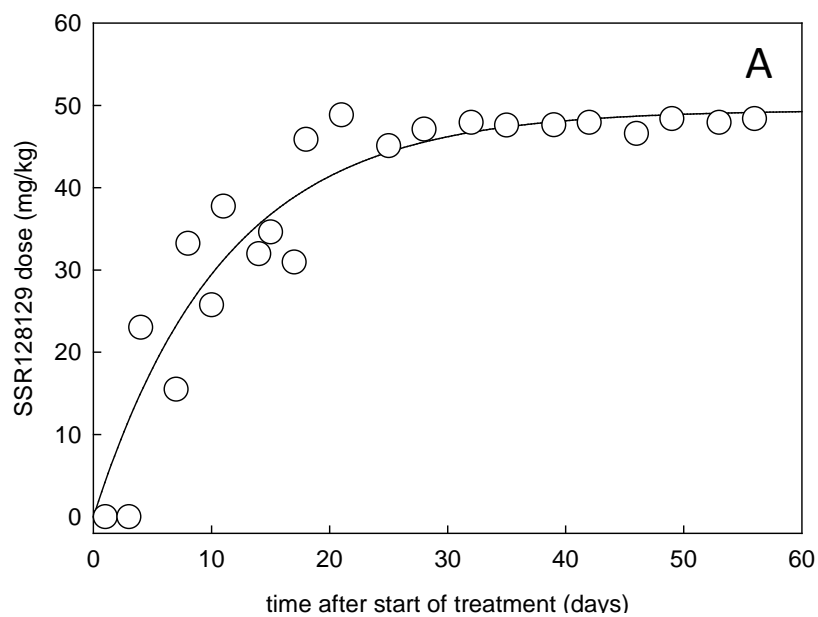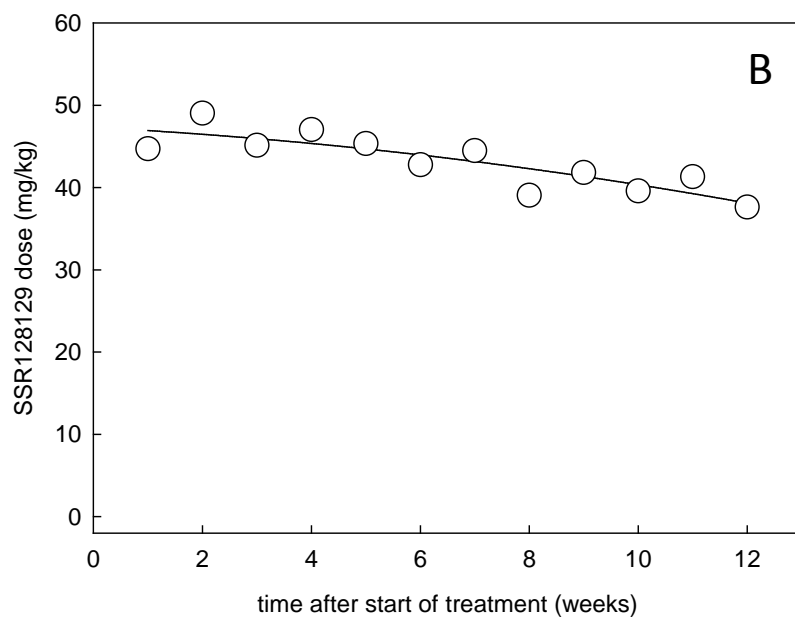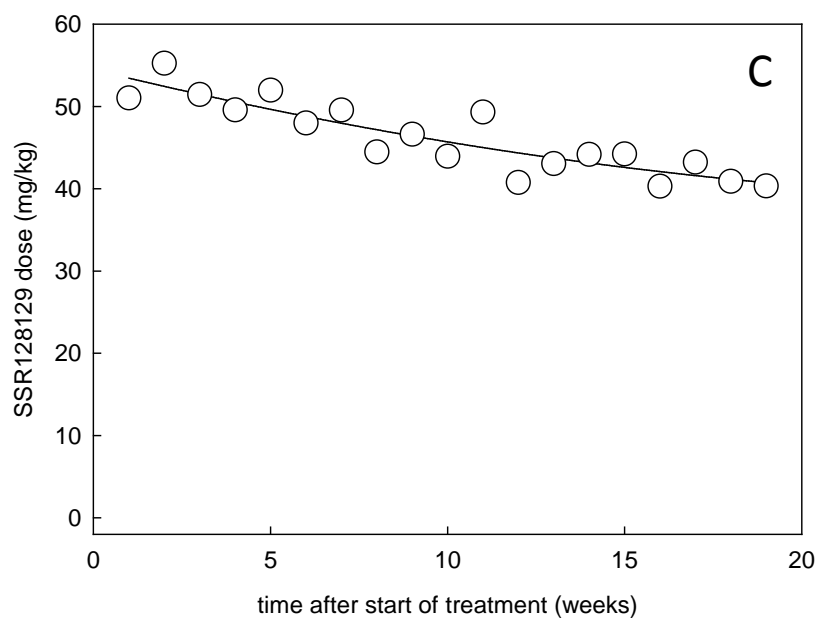

Supplement: Figure S1 — Daily dose of SSR128129E as calculated from food uptake in the different models. The daily dose was determined from the concentration of SSR128129E in the food pellets (367 mg/kg), food intake and animal weight in the vein graft model (A) and during 3 month (B) and 5 month (C) treatment in apoE-deficient mice. (PDF) [file pone.0080027.s001.pdf]
